# Supplementary material for: One Health Approach to the Computational Design of a Lipoprotein-Based Multi-Epitope Vaccine Against Human and Livestock Tuberculosis
Source: Int J Mol Sci. 2025 Feb 13;26(4):1587. doi: 10.3390/ijms26041587 (PMC11855821; doi:10.3390/ijms26041587)
Supplement: Supplementary file 1 [file ijms-26-01587-s001.zip › ijms-3432651-supplementary.pdf]

Supplementary Table S1. Preliminary characterization of selected lipoproteins.

Protein	Localization		Signal Peptide		Transmembrane domains	
	DeepLoc	TBpred	SignalP 6.0	TOPCONS	DeepTMHMM	TOPCONS
P9WIF5	Extracellular	Integral membrane protein	No	Yes (21/22)	0	0
O53692	Golgi apparatus	Secreted protein	No	No	0	0
Q79FB3	Extracellular	Integral membrane protein	No	Yes (21/22)	0	0
P9WIB5	Mitochondrion	Integral membrane protein	Tat (40/41)	Yes (30/31)	0	0
P9WJE1	Cytoplasm	Integral membrane protein	No	No	0	0
**P9WK61**	Extracellular	Protein attached to membrane by lipid anchor	Lip (20/21)	Yes (24/25)	0	0
**P9WK65**	Extracellular	Protein attached to membrane by lipid anchor	Lip (26/27)	Yes (29/30)	0	0
**P9WNF3**	Extracellular	Secreted protein	Lip (24/25)	Yes (27/28)	0	0
**P9WK45**	Extracellular	Protein attached to membrane by lipid anchor	Lip (26/27)	Yes (30/31)	0	0
P9WG29	Endoplasmic reticulum	Cytoplasmic protein	Lip (23/24)	Yes (22/23)	0	0
O53859	Endoplasmic reticulum	Integral membrane protein	No	Yes (24/25)	0	1 (78-98)
**I6Y3P1**	Extracellular	Cytoplasmic protein	Lip (20/21)	Yes (24/25)	0	0

**A5TZX4**	Extracellular	Integral membrane protein	Sec (46/47)	Yes (30/31)	0	0
**P9WGT7**	Extracellular	Protein attached to membrane by lipid anchor	Lip (22/23)	Yes (25/26)	0	0

Supplementary Table S2. Characteristics of predicted conformational epitopes.

Rank	Residues	Residue number	Score
1	A:K541, A:E543, A:G544, A:A545, A:R546	5	0.983
2	A:H674, A:H675, A:H676, A:H677, A:H678, A:H679, A:H680, A:H681	8	0.885
3	A:K407, A:G408, A:P409, A:G410, A:P411, A:G412, A:T413, A:T414, A:G415, A:S416, A:G417, A:E418, A:T419, A:T420, A:T421, A:A422, A:A423, A:G424, A:T425, A:T426, A:A427, A:S428, A:P429, A:G430, A:A431, A:A432, A:S433, A:G434, A:P435, A:K436, A:G437, A:P438, A:G439, A:P440, A:G441, A:E442, A:T443, A:G444, A:D445, A:H446, A:Q447	41	0.851
4	A:V476, A:S477, A:A478, A:Q479, A:A480, A:V481, A:N482, A:Q483, A:G484, A:P485, A:G486, A:P487, A:G488, A:G489, A:G490, A:L491, A:N492, A:S493, A:L494, A:P495, A:L496, A:P497, A:G498, A:T499, A:A500, A:G501, A:H502, A:G503, A:E504	29	0.818
5	A:G547, A:N548, A:D549, A:G550, A:T551, A:S552, A:A553, A:A554, A:A555, A:K556, A:N557, A:T558, A:P559, A:G560, A:S561, A:I562, A:T563, A:Y564	18	0.813
6	A:Y515, A:P516, A:D517, A:S518, A:Q519, A:V520, A:G521, A:T522	8	0.811
7	A:N535, A:S536, A:S537, A:A538, A:Y539, A:P540	6	0.808
8	A:Y264, A:G265, A:L266, A:S267, A:G268, A:C269, A:S270, A:S271, A:N272, A:K273, A:A274, A:A275, A:Y276, A:T277, A:P278	15	0.776
9	A:K523, A:K524, A:L525, A:K526, A:S527, A:G528, A:D529, A:T530, A:I531, A:G532, A:L533, A:K534	12	0.72
10	A:Q60, A:S61, A:F63, A:V65, A:I66, A:L67, A:E68, A:A69, A:A70, A:G71, A:D72, A:K73, A:K74, A:I75, A:G76, A:V77, A:I78, A:K79, A:V80, A:V81, A:R82, A:E83, A:I84, A:V85, A:S86, A:G87, A:L88, A:G89, A:L90, A:K91, A:E92, A:A93, A:K94, A:D95, A:L96, A:V97, A:D98, A:G99, A:A100, A:P101, A:K102, A:P103, A:L104,	68	0.718

	A:L105, A:E106, A:V108, A:A109, A:K110, A:E111, A:A112, A:A113, A:D114, A:E115, A:A116, A:K117, A:A118, A:K119, A:L120, A:E121, A:A122, A:A123, A:G124, A:A125, A:T126, A:V127, A:T128, A:V129, A:K130		
11	A:M1, A:A2, A:K3, A:L4, A:S5, A:T6, A:D7, A:E8, A:L9, A:L10, A:D11, A:A12, A:F13, A:K14, A:E15, A:M16, A:T17, A:E20	18	0.692
12	A:E509, A:I510, A:V511, A:C512, A:S513, A:K514	6	0.689
13	A:G350, A:P351, A:G352, A:P353, A:G354, A:G355, A:N356, A:D357, A:D358, A:N359, A:V360, A:T361, A:G362, A:G363, A:G364, A:A365, A:T366	17	0.644
14	A:R455, A:G456, A:S457, A:G458, A:N459, A:S460, A:G461, A:P462, A:G463, A:P464, A:G465, A:G466	12	0.638
15	A:R280, A:H281, A:C282, A:R284	4	0.632
16	A:K505, A:K506, A:T507, A:Y508	4	0.631
17	A:D23, A:F24, A:K26, A:K27, A:F28, A:E30, A:T31, A:F32	8	0.623
18	A:S588, A:T589, A:I590, A:G591, A:A592, A:G593, A:Q594, A:S595, A:G596, A:L597, A:G598, A:D599, A:N600, A:G601, A:K602, A:K603, A:T604, A:G634, A:D635, A:N636, A:I637, A:K638, A:K639, A:D640, A:G641, A:K642, A:D643, A:Q644, A:F670, A:Q671, A:G672, A:P673	32	0.614
19	A:A48, A:P49, A:A50, A:G51, A:A52, A:A53, A:V54, A:E55, A:A56, A:A57, A:E58	11	0.591
20	A:K325, A:Q326, A:A327, A:G328, A:P329, A:G330, A:P331, A:G332, A:V333, A:R334	10	0.571
21	A:P194, A:T195, A:A196, A:S197, A:D198, A:P199, A:A200	7	0.564
22	A:Y300, A:N301, A:D302, A:D303, A:R304, A:D306, A:F307, A:V308	8	0.554
23	A:N245, A:L246, A:P247, A:V248, A:V249, A:A250	6	0.552
24	A:R145, A:P146, A:Q147, A:G148, A:G149, A:G150, A:S151	7	0.538
25	A:P242, A:A243, A:W244	3	0.524
